# Ecological Impacts of Megaprojects: Species Succession and Functional Composition

**DOI:** 10.3390/plants10112411

**Published:** 2021-11-09

**Authors:** Hamada E. Ali, Solveig Franziska Bucher

**Affiliations:** 1 Botany and Microbiology Department, Faculty of Science, Suez Canal University, Ismailia 41522, Egypt; 2 Department of Biology, College of Science, Sultan Qaboos University, Muscat 123, Oman; 3 Professorship of Plant Biodiversity, Institute of Ecology and Evolution with Herbarium Haussknecht and Botanical Garden, Friedrich Schiller University Jena, D-03743 Jena, Germany; solveig.franziska.bucher@uni-jena.de

**Keywords:** construction sites, invasive plant species, pipeline, plant functional traits, vegetation recovery

## Abstract

Land-use changes have huge impacts on natural vegetation, especially megaprojects, as the vegetation layer is destroyed in the course of construction works affecting the plant community composition and functionality. This large-scale disturbance might be a gateway for the establishment of invasive plant species, which can outcompete the natural flora. In contrast, species occurring in the area before the construction are not able to re-establish. In this study, we analyzed the impact of a pipeline construction on a wetland nature reserve located in northern Egypt. Therefore, we analyzed the plant species occurrence and abundance and measured each plant species’ traits before the construction in 2017 as well as on multiple occasions up to 2 years after the construction had finished on altogether five sampling events. We found that the construction activity led to the establishment of an invasive species which previously did not occur in the area, namely, *Imperata cylindrica*, whereas five species (*Ipomoea carnea*, *Pluchea dioscoridis*, *Polygonum equisetiforme*, *Tamarix nilotica*, and *Typha domingensis*) could not re-establish after the disturbance. The functionality of ecosystems assessed via the analysis of plant functional traits (plant height, specific leaf area, and leaf dry matter content) changed within species over all sampling events and within the community showing a tendency to approximate pre-construction values. Functional dispersion and Rao’s quadratic diversity were higher after the megaproject than before. These findings are important to capture possible re-establishment and recovery of natural vegetation after construction and raise awareness to the impact of megaprojects, especially in areas which are high priority for conservation.

## 1. Introduction

Human activities in the form of changes in land use, such as the intensification of agriculture, fragmentations of natural habitats through extraction of natural resources, or construction works which lead to a widespread removal of natural vegetation, as well as sealing of surfaces, have contributed to a global decline in biodiversity [1,2,3]. A continuous land-use history with little changes in the degree of disturbance is not only important for the conservation of typical cultural landscapes [4,5] and the species within [6,7], but also for ecosystem services such as nutrient cycling, provision of stable habitat for individual species, and successful pollination of plants [8,9]. A decline of biodiversity in response to land-use changes is well documented [10,11]. The impact of agriculture has been the focus of many research projects, yet the direct impact of megaprojects [12] on natural vegetation has not been studied thoroughly. As human impact has a huge influence on vegetation composition and biodiversity via construction activities, the possibility of the return of naturally occurring vegetation to the original state after a megaproject remains unclear.

One of the most fundamental changes in natural vegetation in the course of megaprojects is the introduction of new species, which have not occurred in the area before. On the one hand, species get introduced to the area via the transportation of seeds by different seed dispersal methods. On the other hand, competition is reduced by the disturbance, and thus, new species already present in the area can successfully establish. Due to global trade and transportation, the spread of non-native species, which sometimes become invasive in their non-natural ranges, is getting more rapid. Such invasions by alien species can have major environmental and economic impacts [13,14]. The ability of non-native species to establish in new habitats outside their native ranges is often linked to reduced competition and the occupation of previously unoccupied niches. Most new arrivals have broader ecophysiological niches or experience a reduced herbivore pressure as compared to native species [14,15,16]. 

One easy tool for measuring plant properties is the assessment of plant functional traits which offer the possibility to analyze a species’ (eco-)physiological properties and its functions within the ecosystem [17,18,19,20,21]. Important parameters to assess these are maximum plant height (H_max_), which indicates the competitive strength of a species mainly for better light assessment [17,22,23], specific leaf area (SLA), as high SLA is linked to high growth rates, short leaf longevity and poor nutrient conservation strategies of a species [24,25,26,27], and leaf dry matter content (LDMC), a proxy of the investment in structural compounds which indicates tougher, long lived leaves and good defense [17]. Traits of the new arrivals are either dissimilar to native species allowing them to occupy different niches or similar if abiotic conditions are extreme and/or only a certain set of traits can give competitive advantages and allow a species to pass the environmental filter to be accepted in the local species pool [28,29,30,31,32]. Furthermore, the variability within species might change due to the huge disturbance and the necessary re-establishment. Thus, they have the power to change the overall ecosystem functioning and properties that can be defined by community-weighted mean values. For restoration and re-establishment of natural communities, establishing the functionality of natural communities, not so much the species composition, can be a conservation target [33,34]. It is not clear whether megaprojects and their consequences will not only change plant species abundance and occurrence but also alter the community trait values and thus the function of populations before and after construction work, and whether incoming invasive species can play a part in this.

To study the effects of a megaproject on the natural vegetation in both its composition and functioning, we studied the impact of a pipeline construction site. In this study, we focus on wetland landscapes, located at the border in between the sea and the land situated within the Lake Manzala protected area in the northern coastal part of Egypt. The vegetation there provides a corridor that has an important function for conservation [35,36], as it provides species refuges as well as feeding and breeding habitats for animals [37] and soil erosion control [38,39,40]. The area is facing major disturbances due to the construction of the pipeline which eliminated the whole vegetation of the area as the native soil has been removed, and not necessarily native soil with seeds foreign to the area were brought in at the end of the construction. More specifically, we have asked the following questions:

1. Are plant communities able to regenerate naturally and return to their initial re-establishment trajectory state in terms of species occurrence and abundance after pipeline construction and are there changes in the occurrence and abundance of species classified as invasive?
2. Are there any functional differences between the established vegetation and the original one before the megaproject?

## 2. Results

### Natural Regeneration after the Construction

We identified a total of 14 plant species in our study, with 4 species being annuals, and the other 10 species were perennials. While the mean cover percentage for the plots before the pipeline construction was 90.4%, it was 3.1, 8, 55.4, and 83.9% in the four measurement events performed after the pipeline construction within the timeframe of the study (df = 206, *F* = 1014, *p* < 0.001) (Figure S2). Of the 14 species, 5 failed to reintroduce in the area after pipeline construction (namely, *Ipomoea carnea* Jacq., *Pluchea dioscoridis* (L.) DC., *Polygonum equisetiforme* Sibth. & Sm., *Tamarix nilotica* (Ehrenb.) Bunge, and *Typha domingensis* Pers.), 2 could not be found in the first year after construction but occurred in the second year (*Halocnemum strobilaceum* (Pall.) Bieb. and *Zygophyllum aegyptium* A. Hosny), whereas 1 species, namely, the invasive *Imperata cylindrica* (L.) P. Beauv., occurred only after the megaproject. Before the construction, there was only one species that was categorized as invasive, *Ipomoea carnea,* but it failed to regenerate after the project construction (for cover percentage on species level, see Figure S3).

**Figure 1:**
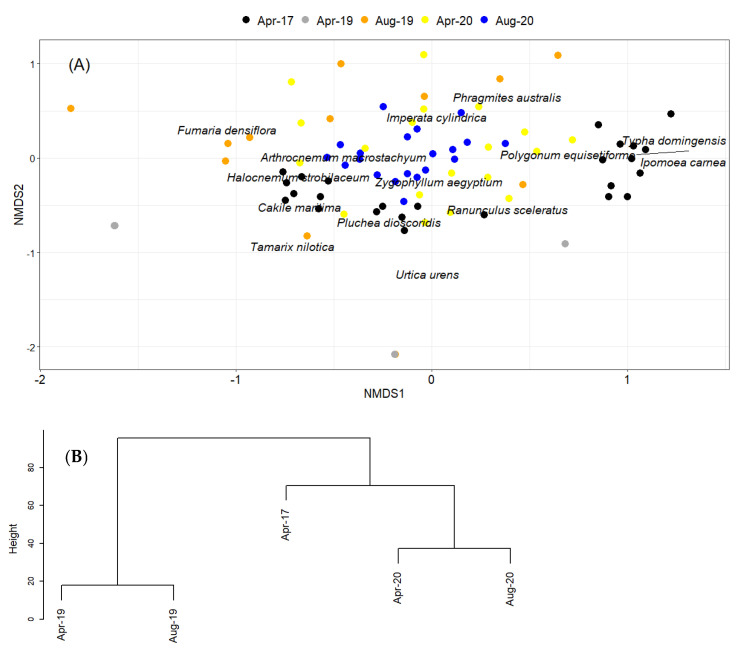
(**A**) NMDS ordination of plots at different sampling events (displayed by colors) and the species recorded during the surveys. (**B**) Hierarchical clustering of plots at different sampling events.

Nonmetric multidimensional scaling ordination (NMDS) resulted in a 2-dimensional configuration that described 81% of the variation in the plot matrix in terms of species abundance (Figure 1A). NMDS analysis confirmed that plant species composition differed significantly between the pre- and post-pipeline construction (*R*^2^ = 0.34, *p* < 0.001; Figure 1A). However, the 2017 samples are unique in separating into two distinct clusters. The cluster characterized by *Typha domingensis* (i.e., with large positive loading on NMDS axis 1, Figure 1A) has not re-established in post-pipeline samples. Moreover, the hierarchical analysis of plots before and after the pipeline construction showed a distinct separation between 2019 and 2020 plots, with the 2020 plots being more similar to the state before the construction than the plots in 2019, the year after construction (Figure 1B).

**Figure 2:**
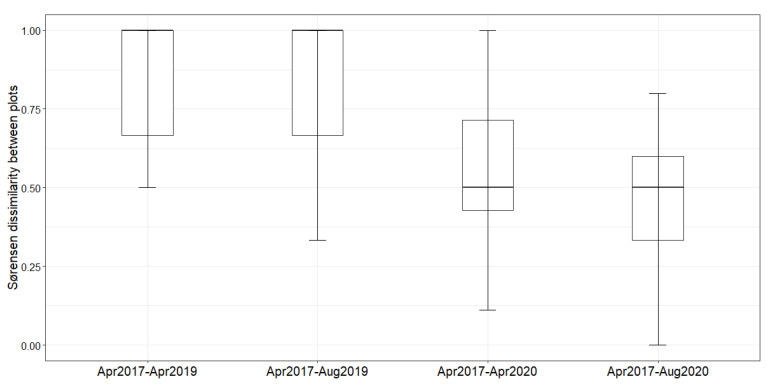
Sørensen pairwise dissimilarity (*β_sor_*) between pre-pipeline (April 2017) and after (April 2019, August 2019, April 2020, and August 2020) pipeline construction on functional traits at the species and community level.

The Sørensen pairwise dissimilarity (*β_sor_*) showed that the plots sampled in 2020 are less dissimilar in comparison to the plots sampled in 2019 (Figure 2).

Most of the species recorded during the surveys tend to decrease their H_max_ and LDMC values immediately after the construction, followed by a steady increase over time back to (almost) pre-construction conditions (Figure S4, Table S1). In turn, SLA showed an opposite behavior as it sharply increased after the construction and then decreased (Figure S4, Table S1). H_max_ increased in *Arthrocnemum macrostachyum* (Moric.) C. Koch., *Cakile maritima* Scop., and *Ranunculus sceleratus* L. back to their original values but not in in *Fumaria densiflora* DC., *H. strobilaceum*, *Phragmites australis* (Cav.) Trin. ex Steud., and *Urtica urens* L., which nevertheless show increased H_max_ compared with immediately after the construction (Figure S4A). *Zygophyllum aegyptium* was even higher after pipeline construction than before. *Imperata cylindrica* constantly increased H_max_ and LDMC after introduction. For statistical analysis, please see Table S1. For LDMC only, *C. maritima* reached its original values whereas the other species had lower values than before (Figure S4C, for statistical details see Table S1). The same behavior was noticed in the SLA, in which *A. macrostachyum, R. sceleratus*, and *P. australis* reached their original values, whereas *U. urens* and *Z. aegyptium* had lower values in comparison to values before the pipeline construction (Figure S4B, for statistical details see Table S1). Moreover, *I. cylindrica* constantly decreased in terms of SLA values after introduction.

**Figure 3:**
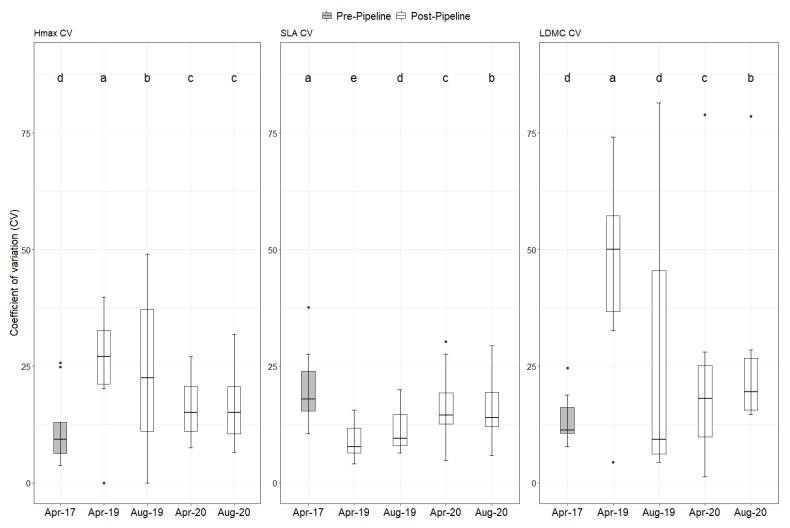
Coefficient of variation (cv) of the maximum plant height (H_max_), specific leaf area (SLA), and leaf dry matter content (LDMC) at each of the five sampling events (before and after the project). Letters above the boxes indicate significant differences based on Tukey’s HSD at the *p* < 0.05 threshold.

The variability of the plant functional traits revealed that in both H_max_ and LDMC the pre-pipeline plots were less variable when compared to the post-pipeline construction (Figure 3). On the contrary, for SLA, the pre-pipeline plots were more variable in comparison to the post-pipeline plots (Figure 3, for details see Table S2).

**Figure 4:**
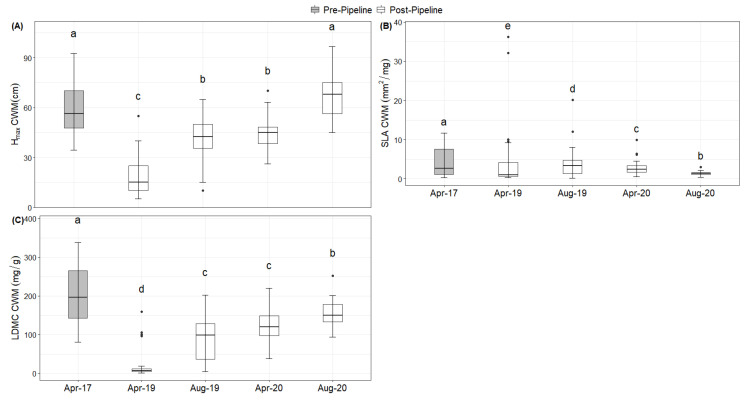
Differences in the community-weighted means of the plant functional traits for the five sampling events (before and after the megaproject) in (**A**) maximum plant height (H_max_), (**B**) specific leaf area (SLA), and (**C**) leaf dry matter content (LDMC). Letters above the boxes indicate significant differences based on Tukey’s HSD at the *p* < 0.05 threshold.

The community-weighted means were higher before and after the megaproject for H_max_ (df = 206, *F* = 68.15, *p* < 0.001; Figure 4A) and LDMC (df = 206, *F* = 55.21, *p* < 0.001, Figure 4C), yet they were lower for SLA (df = 206, *F* = 27.36, *p* < 0.001, Figure 4B). However, minimum values of H_max_ and LDMC were found immediately after the construction and increased over time. For SLA, however, it had higher values immediately after the construction and decreased afterwards.

**Figure 5:**
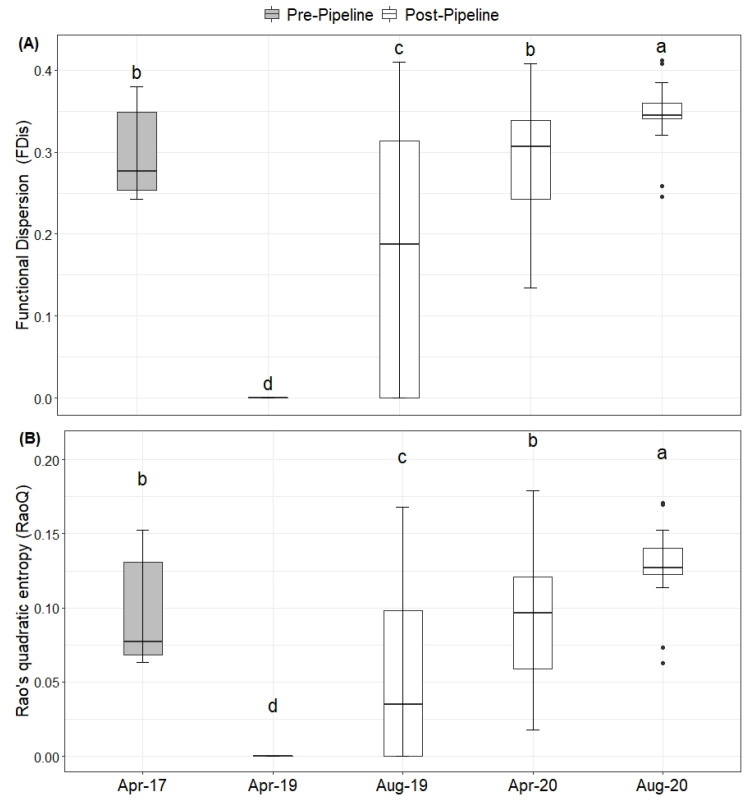
Functional diversity indices for the five sampling events (before and after the project) in (**A**) functional dispersion (FDis) and (**B**) Rao’s quadratic entropy (RaoQ). Letters above the boxes indicate significant differences based on Tukey’s HSD at the *p* < 0.05 threshold.

For FDis (df = 206, *F* = 113.5, *p* < 0.001; Figure 5A) and RaoQ (df = 206, *F* = 79.05, *p* < 0.001; Figure 5B), we noticed an increase in both indices for the last sampling event (August 2020) in comparison to the initial sampling event that was conducted before the construction of the pipeline (April 2017). Again, the lowest values were found immediately after the construction, but a gradual increase could be detected.

## 3. Discussion

In this paper, we studied the consequences of a megaproject on the natural vegetation of a wetland site of high conservation priority. We evaluated the re-establishment trajectory of plant species after this major disturbance by analyzing their occurrence and abundance and the functional compositions of the plant communities before and after the construction. We found that not all plant species could re-establish after the megaproject within the first two years after the construction. By contrast, an invasive species (*Imperata cylindrica*) was introduced into the study system. On an intraspecific scale, we could detect that species changed their trait values but had the tendency to reach their original values in H_max_, SLA, and LDMC over time, which was also true for community-weighted means. FDis and RaoQ were even higher after the pipeline construction.

Regeneration of the natural vegetation was thus only partially possible after the major disturbance and happened slowly. The construction posed a major disruption of the natural conditions as the soil was dug up to 5 m deep and 5 m wide and partially soil which did not originate from the study area was introduced into the system, which was not sterilized. Therefore, seeds from outside the study region were distributed, facilitating the establishment of new species and maybe changing soil nutrient conditions. However, after the megaproject, we only observed eight species from the thirteen founded before the construction, which represents an overall species loss of 30.8% This is in line with other research which demonstrated species loss in areas with high changes in land-use regimes and huge disturbances [10,11]. However, temporal development in vegetation was apparent, where the composition of the plant communities of the 2020 sampling event went more toward the original communities of the 2017 sampling event (as in Figure 1B). Nevertheless, this similarity is limited because of the lack of re-establishment of communities associated with *Typha domingensis* and *Polygonum equisetiforme* (Figure 1A). The NMDS analysis showed that the communities were significantly different before and after the construction project, with the original vegetation before the construction forming two distinct types of vegetation. The newly established communities after the construction showed a development getting closer to the original vegetation over time. Yet, there was a clear difference between the two types of vegetation: the sites dominated by *A. macrostachyum* and *Z. aegyptium* almost returned to their natural state based on the NMDS, whereas the ones dominated by *T. domingensis*, *P. australis*, and *I. carnea* are preferred by migratory bird species. This vegetation type did not seem to be able to recover as the keystone species were not able to re-establish. As we analyzed the vegetation only a maximum of 20 months after the end of the megaproject, this might only be a snapshot in time of the initial re-establishment phase of vegetation, as further changes might occur due to the proximity of natural, undisturbed vegetation. Long-term monitoring of the restauration of seasonal wetlands showed that species accumulated and thus diversity strongly increased within the first 12 years after reflooding [41]. 

Note that because we used the 2017 vegetation community as our baseline, we are unable to account for any natural successional change that might have occurred over the period 2017–2020 and therefore assume all differences in composition are attributable to the impacts of pipeline construction.

The megaproject was a gateway to the introduction of alien plant species, as *I. cylindrica* could establish in the system in almost all plots studied after the construction. However, the invasive species *I. carnea* was lost from the local species pool after the megaproject. *I. cylindrica* is a perennial rhizomatous grass of the Poaceae family, which is highly flammable and mainly native to tropical and subtropical Asia [42]. It is considered a weedy pest in 73 countries worldwide. Commonly, most non-native species are drawn to disturbed and/or nutrient rich ecosystems. The introduction of *I. cylindrica* is a worrying development from a conservation perspective due to its spread by rhizome and due to its ability to outcompete smaller species it can form mono-species stands quite quickly [43,44,45]. Moreover, the potentially increased flammability of the study system could pose a problem for birds, especially breeding birds. *I. cylindrica* is a tall species and was only rivalled in height by *A. macrostachyum* and *P. australis* in our study system (Figure S4A), which illustrates its competitive strength [17]. It is quite similar in its trait composition to the already existing species as far as SLA and LDMC are concerned, and thus rather being already optimized to the harsh conditions such as low precipitation and high temperatures [28,29,30,31,32]. Normally, species, according to the limiting similarity hypothesis, need to be dissimilar enough from the already existing vegetation to avoid competition, which leads to trait divergence [34,46,47,48], yet this might have been counterbalanced by the major disturbance event which eradicated the direct competitors. 

The comparably large height of *I. cylindrica* is partly a reason why H_max_, unlike SLA and LDMC of the community, did not change before and after the pipeline construction. However, the highest species of the plots, *T. domingensis*, was lost from the study system, and maybe even replaced in its ecological niche by *I. cylindrica*. While LDMC increased in every sampling event, SLA decreased but did not quite make it to pre-pipeline conditions on a community scale in the first 2 years after construction. This might be due to the fact that *I. carnea*, *P. equisetiforme*, *T. nilotica*, and *T. domingensis* were lost from the systems. These species are high in SLA and/or LDMC. The new species, *I. cylindrica*, is high in LDMC but low in SLA and thus not able to maintain the same level of SLA and LDMC on a community scale. Taking intraspecific variation into account, instead of just measuring plant functional traits once at full flowering as recommended by standardized protocols [17], allowed us to assess changes in community-weighted means, which were due to all plant traits showing a decreased value of H_max_ and LDMC and increased SLA within each species directly after the pipeline construction, when individuals were not fully grown yet. It is well documented that plant traits change within the seasons [49] and respond to changes in abiotic conditions such as temperature [50]. Thus, part of the variation between plant traits sampled at the different sampling events is also due to differences in climate between sampling seasons and the two sampling years. The intraspecific trait variability might also be more important than absolute trait values for the successful recolonization of a given habitat. High variability is often linked to the high capability to adapt to changing environmental conditions [47,51,52]. It is shown here that the communities were much more variable in SLA as before the construction, maybe also indicating some new functional niches which did not exist previously.

## 4. Materials and Methods

### 4.1. Site Description

**Figure 6:**
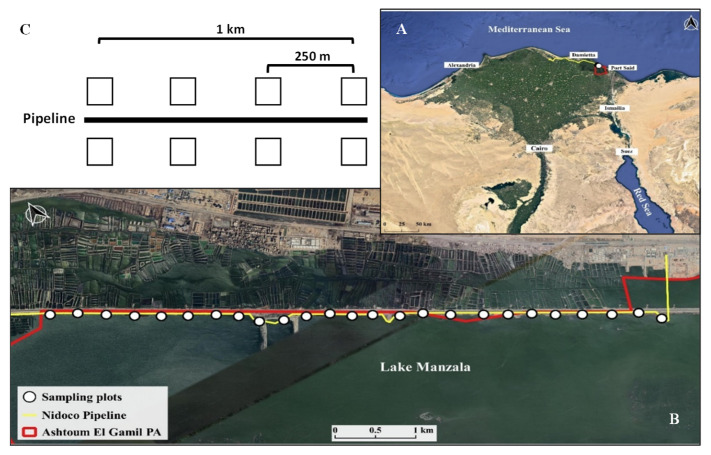
(**A**) Location of the study area showing the Ashtoum El-Gamil protected area, (**B**) the fifty sampling plots over the megaproject, and (**C**) the sampling scheme for the pre- and post-pipeline construction.

This study was conducted at the Lake Manzala coast (31°15′ N, 32°11′ E, 1148 m), located in the northern part of Egypt (Figure 6A). The area has a minimum annual temperature of 12.5 °C during January, while it increases gradually to reach a maximum of 30 °C in August with a total of 100 mm rainfall per year [53]. The relative humidity is fairly constant throughout the year and ranges between 60% and 75%.

The soil of the study area is characterized by its coarse texture with high amounts of sand and gravel due to the sea currents and waves coming from the lake which wash the fine parts away from the shore. It is also characterized by low pH due to the decay of the organic materials and the release of organic acids in soil, and high CaCO_3_ content due to the presence of large amount of shell remnants mixed with the soil and low nitrogen and phosphorus contents [54]. 

In 2018, three pipelines were built connecting an onshore well located in the Governorate of Kafr Elsheikh to the Abu Madi Plant (Eldakhli Governorate) and the El Gamil Plant (Port Said Governorate) passing through the Damieta Governorate in the Nile Delta region. The construction was finished by the end of the year 2018. The identified pipeline corridors mainly affect agricultural areas and fish farms as well as large water bodies. However, the pipelines cross several roads, canals, and rivers and, crucially, Lake Manzala, which is an area of high conservation value designated as an IBA (Important Bird Area) [45] and is in parts designated as a National Protected Area (NPA) [55] (Figure 6B). In 2019, the Egyptian Environmental Authorities modified the perimeter of the Ashtoum El-Gamil Protected Area (Prime Ministerial Decree 2433, 2019). The pipeline is now within the newly defined boundaries of the Protected Area (Figure 6).

### 4.2. Vegetation Surveys

To identify the impact of the newly constructed pipeline on the composition of the plant communities grown within the study area (in terms of plant occurrence and abundance as well as functionality), we have made botanical surveys of 50 plots with the size of 25 m^2^ each located on both sides of the pipeline (Figure 6C). These surveys took place five times, once before the pipeline construction (April 2017) and four after the construction of the pipeline capturing successional processes (April 2019, August 2019, April 2020, and August 2020, to mark the beginning and end of the growing season) to analyze the effects of the megaproject (for photos showing the plots at different sampling events, see Figure S1).

We designed our study to cover the ten kilometers of the pipeline construction site in the Ashtoum El-Gamil protected area (Figure 1). We spaced our plots evenly along these ten kilometers, with each plot having a size of 5 × 5 m (25 m^2^). Plots were placed along the transect every 250 m; in total, 50 plots were studied during the survey (Figure 1), which adds up to a total number of 250 vegetation plots. At each of the plots mentioned above and at every sampling event, we identified all plant species present, recorded the individual cover percentage of each plant grown, assessed the total vegetation cover percentage of the entire plot, and calculated the species richness thereof [56]. Moreover, we calculated the Sørensen pairwise dissimilarity (*β_sor_*) for each plot with itself at different sampling events (i.e., April 2017 and April 2019; April 2017 and August 2019; April 2017 and April 2020; and April 2017 and August 2020). Here, we assume that our baseline is the pre-pipeline construction (April 2017). We calculated the *β_sor_* using the following equation:

(1) $$\beta_{sor}=\;\frac{b+c}{2a+b+c}$$

where *β_sor_* is Sørensen dissimilarity, *a* is the number of species present in both plots, *b* is the number of species present only in plot *X*, and *c* is the number of species present only in plot *Y*, such that *a* + *b* + *c* is the total number of species in the two plots [57].

### 4.3. Plant Functional Traits and Community Characteristics

Plant functional traits, community-weighted mean (CWM), and diversity parameters to characterize the functional composition of the communities were assessed on each plot on each of the sampling events. We performed plant functional trait measures on healthy, fully grown individuals in each plot on all occurring plant species. We measured H_max_, SLA, and LDMC on five individuals per species per plot. H_max_ was determined as the shortest distance from ground level to the highest photosynthetic tissue using a meter. To measure SLA and LDMC, five healthy fully developed and sun-exposed leaves were collected for each of the five individuals and measured together as one pooled sample. We measured SLA, defined as the ratio of fresh leaf area (LA) to dry mass expressed as mm^2^ mg^−1^; the two leaf dimensions were measured manually using a ruler (mm), then these two dimensions were multiplied to obtain the LA (mm^2^), the leaves were oven-dried at 70 °C for 48 h, and subsequently weighed with a precision of 1 μg to obtain the leaf dry mass (mg). Finally, the LA was divided by the leaf dry matter to calculate SLA. In addition to that, we measured the leaf dry matter content (LDMC) as the oven-dry mass (mg) of a leaf, divided by its water-saturated fresh mass (g), expressed in mg g^−1^. All plant functional traits were measured based on the standard methods developed by [17]. To estimate the variability of the three selected traits between the sampling dates, we calculated the coefficient of variation (cv) for each species at each sampling event and expressed it as a percentage.

Functional properties of the individual species on every plot were then used to calculate several functional community values to compare the communities between the five measurement events (before and after the project implementation), namely, the community-weighted means (CWM), the functional dispersion (FDis), and Rao’s quadratic diversity (RaoQ). The CWMs for each of the traits was calculated separately [18,58,59] using the following equation:

(2) $$CWM=\sum_{i=1}^{n}p_{i}\;trait_{i}$$

where *n* is the number of species in the community for every measurement event, *p_i_* is the species cover of the given species *i* in each plot and *trait_i_* is the mean of species-specific trait values (one value per trait, plot, and measurement event). We also calculated FDis, which describes the abundance-weighted mean distance of individual species to their group centroid in a multivariate trait space following the method described by Laliberté and Legendre [60]:

(3) $$c=\sum \left(a_{j}x_{ij}\right)/\sum a_{j}$$

where *c* is the weighted centroid in the *i*-dimensional space, *a_j_* is the abundance of species *j*, and *x_ij_* is the attribute of species *j* for trait *i*. FDis, the weighted mean distance *z* to the weighted centroid *c*, is then computed as

(4) $$FDis=\sum \left(a_{i}z_{j}\right)/\sum a_{j}$$

where *a_j_* is the abundance of species *j* and *z_j_* is the distance of species *j* to the weighted centroid *c*. Additionally, we calculated the RaoQ [61], which is calculated as the sum of pairwise functional distances between species weighted by their relative abundances [62,63]:

(5) $$RaoQ=\sum_{i=1}^{n}\sum_{j=1}^{n}p_{i}p_{j}p_{ij}$$

where *n* is the number of species in the sample, *p_i_* is the relative abundance of species *i*, *p_j_* is the relative abundance of species *j*, and *p_ij_* describes the functional dissimilarity between species *i* and *j*.

### 4.4. Statistical Analyses

To investigate how the community composition changed due to the pipeline construction, we used nonmetric multidimensional scaling (NMDS) ordination of abundance data for the species recorded in the 50 plots during the five measuring campaigns to visualize variation in the species composition before and after the pipeline construction [64].

To analyze the difference of functional traits before and after pipeline construction, we looked at the intraspecific scale assessing how plant species change their trait values (H_max_, SLA, LDMC) between the five sampling times as well as the variability of trait values assessed by the coefficient of variation (cv), which was performed by calculating the cv for each species at each sampling event for each trait. To assess changes in the functionality of the communities, we calculated CWMs of all traits, FDis and RaoQ for each sampling date and plot, and analyzed how that changed between sampling dates. To analyze the differences between sampling dates within species and the community scale, we used ANOVAs followed by Tukey’s HSD to test using the trait value or the community trait value as the dependent variable and the sampling event as the explanatory variable [65]. 

All these analyses were performed using R, version 4.0.2 [66]. The NMDS was undertaken using the package *vegan* [67]. The functional diversity (FDis and RaoQ) was calculated with the package *FD* [68]. The Sørensen pairwise dissimilarity (*β_sor_*) was calculated using *betapart* package [57]. Model requirements and assumptions were checked and fulfilled for all ANOVA analyses, as variances were homogeneous and residuals normally distributed. 

## 5. Conclusions and Recommendations

We could demonstrate that the natural vegetation can only partially recover from major disturbance events such as megaprojects, at least within the first two years after construction. The introduction of *I. cylindrica* is a worrying development given the detrimental impact it had on previous ecosystems it invaded. However, the invasive species *I. carnea* was lost from the local species pool. From the two types of vegetation occurring in the area prior to the megaproject, only one could regenerate, whereas the other, which is important for breeding birds, could not be due to species loss, which means the regeneration of natural habitats using aided re-establishment via planting should be considered. Functionally, *I. cylindrica* seems to replace the tall species *Typha domingensis* and *Phragmites australis*, so the functionality of the ecosystem remains unchanged. Further observations on how the vegetation develops after a long time span are highly recommendable, though, to assess long-term effects and further species establishments.

## Data Availability

Raw data and R codes are available from the author (H.E.A).

## Supplementary Materials

The following are available online at https://www.mdpi.com/article/10.3390/plants10112411/s1, Figure S1. Differences in vegetation cover before and after the pipeline construction where (A) shows the vegetation cover before the pipeline construction (April 2017); (B) April 2019; (C) August 2019; (D) April 2020; and (E) August 2020, Figure S2: Differences in cover percentage between the five sampling events (pre-pipeline construction in grey and post-pipeline construction in white). Letters above the boxes indicate significant differences based on Tukey’s HSD at the *p* < 0.05 threshold, Figure S3: Differences in total species cover percentages per plot for the five sampling events (before and after the megaproject), Figure S4: Differences of plant functional traits between species for the five sampling events (before and after the project) in (A) maximum plant height (H_max_), (B) specific leaf area (SLA), and (C) leaf dry matter content (LDMC), Table S1: ANOVA output for differences in maximum plant height (H_max_), specific leaf area (SLA), and leaf dry matter content (LDMC) between the 14 recorded species for the five sampling events, Table S2: The coefficient of variation (cv) of the maximum plant height (H_max_), specific leaf area (SLA), and leaf dry matter content (LDMC) for each species at each of the five sampling events (before and after the project).

## References

[1] Fritch R.A., Sheridan H., Finn J., Kirwan L., Huallacháin D.Ó. (2011). Methods of enhancing botanical diversity within field margins of intensively managed grassland: A 7-year field experiment. J. Appl. Ecol..

[2] Newbold T., Hudson L., Hill S.L.L., Contu S., Lysenko I., Senior R., Borger L., Bennett D.J., Choimes A., Collen B. (2015). Global effects of land use on local terrestrial biodiversity. Nat. Cell Biol..

[3] Van Kleunen M., Dawson W., Essl F., Pergl J., Winter M., Weber E., Kreft H., Weigelt P., Kartesz J., Nishino M. (2015). Global Exchange and Accumulation of Non-Native Plants. Nature.

[4] Berkes F., Davidson-Hunt I.J. (2006). Biodiversity, Traditional Management Systems, and Cultural Landscapes: Examples from the Boreal Forest of Canada. Int. Soc. Sci. J..

[5] Gao H., Ouyang Z., Chen S., Van Koppen C.S.A. (2013). Role of culturally protected forests in biodiversity conservation in Southeast China. Biodivers. Conserv..

[6] Myers N., Mittermeier R.A., Mittermeier C.G., Da Fonseca G.A., Kent J. (2000). Biodiversity Hotspots for Conservation Priorities. Nature.

[7] Pimm S.L., Jenkins C.N., Abell R., Brooks T.M., Gittleman J.L., Joppa L.N., Raven P.H., Roberts C.M., Sexton J.O. (2014). The biodiversity of species and their rates of extinction, distribution, and protection. Science.

[8] Naeem S., Duffy J.E., Zavaleta E. (2012). The Functions of Biological Diversity in an Age of Extinction. Science.

[9] Reich P.B., Tilman D., Isbell F., Mueller K., Hobbie S.E., Flynn D.F., Eisenhauer N. (2012). Impacts of Biodiversity Loss Escalate through Time as Redundancy Fades. Science.

[10] Chapin F.S., Zavaleta E.S., Eviner V.T., Naylor R.L., Vitousek P.M., Reynolds H.L., Hooper D.U., Lavorel S., Sala O.E., Hobbie S.E. (2000). Consequences of Changing Biodiversity. Nature.

[11] Sala O.E., Chapin F.S., Armesto J.J., Berlow E., Bloomfield J., Dirzo R., Huber-Sanwald E., Huenneke L.F., Jackson R.B., Kinzig A. (2000). Global Biodiversity Scenarios for the Year 2100. Science.

[12] Flyvbjerg B. (2017). The Oxford Handbook of Megaproject Management.

[13] Vilà M., Basnou C., Pyšek P., Josefsson M., Genovesi P., Gollasch S., Nentwig W., Olenin S., Roques A., Roy D. (2010). How Well Do We Understand the Impacts of Alien Species on Ecosystem Services? A Pan-European, Cross-Taxa Assessment. Front. Ecol. Environ..

[14] Higgins S.I., Richardson D. (2014). Invasive plants have broader physiological niches. Proc. Natl. Acad. Sci. USA.

[15] Cappuccino N., Carpenter D. (2005). Invasive exotic plants suffer less herbivory than non-invasive exotic plants. Biol. Lett..

[16] Carboni M., Münkemüller T., Gallien L., Lavergne S., Acosta A.T.R., Thuiller W. (2013). Darwin’s naturalization hypothesis: Scale matters in coastal plant communities. Ecography.

[17] Perez- Harguindeguy N., Díaz S., Garnier E., Lavorel S., Poorter L., Jaureguiberry P., Bret-Harte M.S., Cornwell W., Craine J., Gurvich D.E. (2013). New handbook for standardised measurement of plant functional traits worldwide. Aust. J. Bot..

[18] Violle C., Navas M.L., Vile D., Kazakou E., Fortunel C., Hummel I., Garnier E. (2007). Let the Concept of Trait Be Functional!. Oikos.

[19] De Bello F., Lavorel S., Díaz S., Harrington R., Cornelissen J.H.C., Bardgett R.D., Berg M.P., Cipriotti P., Feld C.K., Hering D. (2010). Towards an assessment of multiple ecosystem processes and services via functional traits. Biodivers. Conserv..

[20] Römermann C., Bernhardt-Römermann M., Kleyer M., Poschlod P. (2009). Substitutes for grazing in semi-natural grasslands—Do mowing or mulching represent valuable alternatives to maintain vegetation structure?. J. Veg. Sci..

[21] Breitschwerdt E., Jandt U., Bruelheide H. (2014). Do newcomers stick to the rules of the residents? Designing trait-based community assembly tests. J. Veg. Sci..

[22] Westoby M., Falster D.S., Moles A.T., Vesk P.A., Wright I.J. (2002). Plant Ecological Strategies: Some Leading Dimensions of Variation Between Species. Annu. Rev. Ecol. Syst..

[23] Gaudet C.L., Keddy P.A. (1988). A comparative approach to predicting competitive ability from plant traits. Nat. Cell Biol..

[24] Lavorel S., Garnier E. (2002). Predicting changes in community composition and ecosystem functioning from plant traits: Revisiting the Holy Grail. Funct. Ecol..

[25] Garnier E. (1992). Growth Analysis of Congeneric Annual and Perennial Grass Species. J. Ecol..

[26] Poorter H., Remkes C. (1990). Leaf area ratio and net assimilation rate of 24 wild species differing in relative growth rate. Oecologia.

[27] Poorter H., Niinemets Ü., Poorter L., Wright I.J., Villar R. (2009). Causes and consequences of variation in leaf mass per area (LMA): A meta-analysis. New Phytol..

[28] Kraft N.J.B., Godoy O., Levine J. (2015). Plant functional traits and the multidimensional nature of species coexistence. Proc. Natl. Acad. Sci. USA.

[29] Diaz S., Cabido M., Casanoves F. (1998). Plant functional traits and environmental filters at a regional scale. J. Veg. Sci..

[30] Keddy P.A. (1992). Assembly and response rules: Two goals for predictive community ecology. J. Veg. Sci..

[31] Venn S.E., Green K., Pickering C.M., Morgan J.W. (2011). Using plant functional traits to explain community composition across a strong environmental filter in Australian alpine snowpatches. Plant Ecol..

[32] Weiher E., Keddy P.A. (1995). Assembly Rules, Null Models, and Trait Dispersion: New Questions from Old Patterns. Oikos.

[33] Engst K., Baasch A., Erfmeier A., Jandt U., May K., Schmiede R., Bruelheide H. (2016). Functional community ecology meets restoration ecology: Assessing the restoration success of alluvial floodplain meadows with functional traits. J. Appl. Ecol..

[34] Laughlin D.C., Joshi C., van Bodegom P., Bastow Z.A., Fulé P.Z. (2012). A predictive model of community assembly that incorporates intraspecific trait variation. Ecol. Lett..

[35] Marshall E.J.P. (1988). The Ecology and Management of Field Margin Floras in England. Outlook Agric..

[36] Moonen A.C., Marshall E. (2001). The influence of sown margin strips, management and boundary structure on herbaceous field margin vegetation in two neighbouring farms in southern England. Agric. Ecosyst. Environ..

[37] Ma M., Hietala R., Kuussaari M., Helenius J. (2013). Impacts of edge density of field patches on plant species richness and community turnover among margin habitats in agricultural landscapes. Ecol. Indic..

[38] Peres G., Cluzeau D., Menasseri-Aubry S., Soussana J.-F., Bessler H., Engels C., Habekost M., Gleixner G., Weigelt A., Weisser W. (2013). Mechanisms linking plant community properties to soil aggregate stability in an experimental grassland plant diversity gradient. Plant Soil.

[39] Pohl M., Alig D., Körner C., Rixen C. (2009). Higher plant diversity enhances soil stability in disturbed alpine ecosystems. Plant Soil.

[40] Ali H.E., Reineking B., Münkemüller T. (2016). Effects of plant functional traits on soil stability: Intraspecific variability matters. Plant Soil.

[41] Aronson M., Galatowitsch S. (2008). Long-term vegetation development of restored prairie pothole wetlands. Wetlands.

[42] Macdonald G.E. (2004). Cogongrass (*Imperata cylindrica*)—Biology, Ecology, and Management. Crit. Rev. Plant Sci..

[43] Shaltout K., Galal T.M., El-Komi T.M. (2016). Phenology, biomass and nutrients of *Imperata cylindrica* and *Desmostachya bipinnata* along the water courses in Nile Delta, Egypt. Rend. Lincei.

[44] Mullié W.C., Meininger P.L. (1983). Waterbird trapping and hunting in Lake Manzala, Egypt, with an outline of its economic significance. Biol. Conserv..

[45] Bird Life International Country Profile: Egypt. http://www.birdlife.org/datazone/country/egypt.

[46] MacArthur R.H., Wilson E.O. (1967). The Theory of Island Biogeography.

[47] Helm J., Dutoit T., Saatkamp A., Bucher S.F., Leiterer M., Römermann C. (2019). Recovery of Mediterranean steppe vegetation after cultivation: Legacy effects on plant composition, soil properties and functional traits. Appl. Veg. Sci..

[48] Wilson J.B. (2007). Trait-Divergence Assembly Rules Have Been Demonstrated: Limiting Similarity Lives! A Reply to Grime. J. Veg. Sci..

[49] Römermann C., Bucher S.F., Hahn M., Bernhardt-Römermann M. (2016). Plant functional traits—Fixed facts or variable depending on the season?. Folia Geobot..

[50] Rosbakh S., Römermann C., Poschlod P. (2015). Specific leaf area correlates with temperature: New evidence of trait variation at the population, species and community levels. Alp. Bot..

[51] Albert C.H., Grassein F., Schurr F.M., Vieilledent G., Violle C. (2011). When and how should intraspecific variability be considered in trait-based plant ecology?. Perspect. Plant Ecol. Evol. Syst..

[52] Jung V., Violle C., Mondy C., Hoffmann L., Muller S.D. (2010). Intraspecific variability and trait-based community assembly. J. Ecol..

[53] Ahmed M.H., El Leithy B.M., Thompson J.R., Flower R.J., Ramdani M., Ayache F., Hassan S.M. (2009). Application of remote sensing to site characterisation and environmental change analysis of North African coastal lagoons. Hydrobiologia.

[54] Elnaggar A.A., El-Alfy M.A. (2016). Physiochemical Properties of Water and Sediments in Manzala Lake, Egypt. J. Environ. Sci..

[55] El Kafrawy S.B., Ahmed M.H., Elbeih S.F., Negm A.M., Kostianoy A. (2020). Monitoring and Protection of Egyptian Northern Lakes Using Remote Sensing Technology. Environmental Remote Sensing in Egypt.

[56] Zahran M.A., Willis A.J. (2008). The Vegetation of Egypt.

[57] Baselga A., Orme C.D.L. (2012). Betapart: An R package for the study of beta diversity. Methods Ecol. Evol..

[58] Lavorel S., Grigulis K., McIntyre S., Williams N., Garden D., Dorrough J., Berman S., Quétier F., Thébault A., Bonis A. (2007). Assessing functional diversity in the field—Methodology matters!. Funct. Ecol..

[59] Garnier E., Lavorel S., Ansquer P., Castro H., Cruz P., Dolezal J., Eriksson O., Fortunel C., Freitas H., Golodets C. (2007). Assessing the Effects of Land-Use Change on Plant Traits, Communities and Ecosystem Functioning in Grasslands: A Standardized Methodology and Lessons from an Application to 11 European Sites. Ann. Bot..

[60] Laliberté E., Legendre P. (2010). A distance-based framework for measuring functional diversity from multiple traits. Ecology.

[61] Rao C. (1982). Diversity and dissimilarity coefficients: A unified approach. Theor. Popul. Biol..

[62] Leps J., de Bello F., Lavorel S., Berman S. (2006). Quantifying and Interpreting Functional Diversity of Natural Communities: Practical Considerations Matter. Preslia.

[63] Ricotta C., Moretti M. (2011). CWM and Rao’s quadratic diversity: A unified framework for functional ecology. Oecologia.

[64] Zar J.H. (1999). Biostatistical Analysis.

[65] Crawley M.J. (2012). The R Book.

[66] (2021). R: A Language and Environment for Statistical Computing.

[67] Vegan: Community Ecology Package R Package, Version 2.5–7. https://CRAN.R-project.org/package=vegan.

[68] Laliberté E., Legendre P., Shipley B. (2014). FD: Measuring Functional Diversity from Multiple Traits, and Other Tools for Functional Ecology. https://www.researchgate.net/publication/312463190_FD_Measuring_functional_diversity_from_multiple_traits_and_other_tools_for_functional_ecology.

