# Supplementary material for: Ecological Impacts of Megaprojects: Species Succession and Functional Composition
Source: Plants (Basel). 2021 Nov 9;10(11):2411. doi: 10.3390/plants10112411 (PMC8622277; doi:10.3390/plants10112411)
Supplement: Supplementary file 1 [file plants-10-02411-s001.zip › plants-1430863-supplementary.pdf]

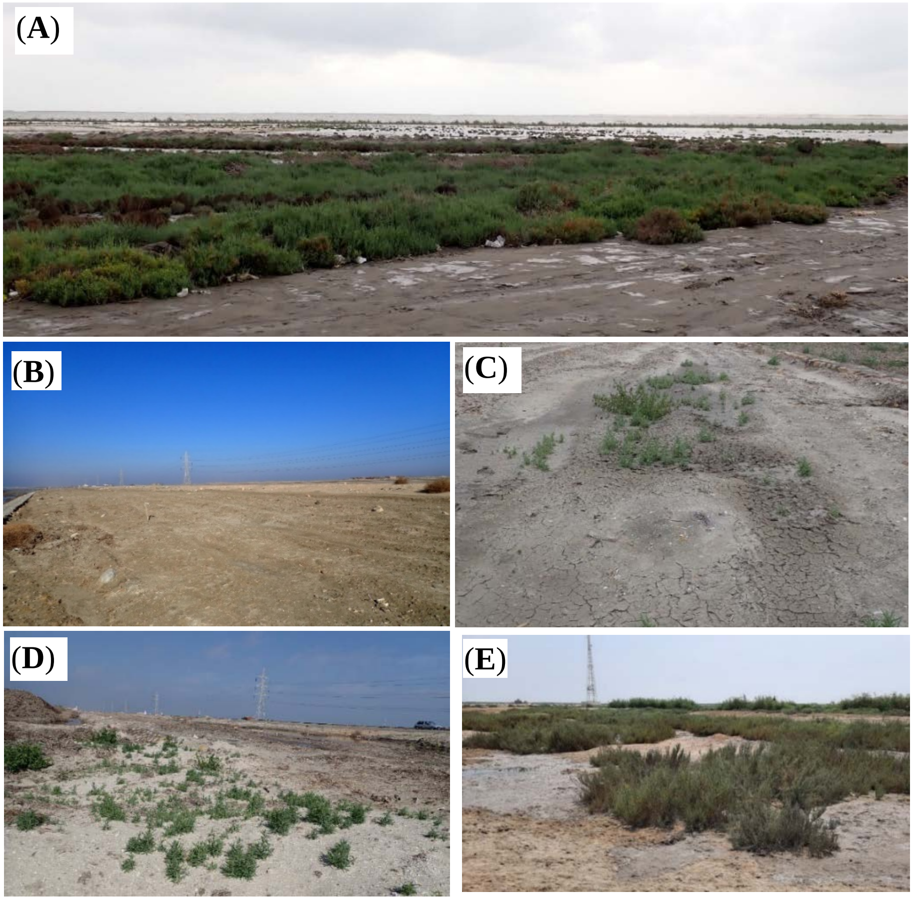

Figure S1. Differences in vegetation cover before and after the pipeline construction where (**A**) shows the vegetation cover before the pipeline construction (April 2017); (**B**) April 2019; (**C**) August 2019; (**D**) April 2020; and (**E**) August 2020.

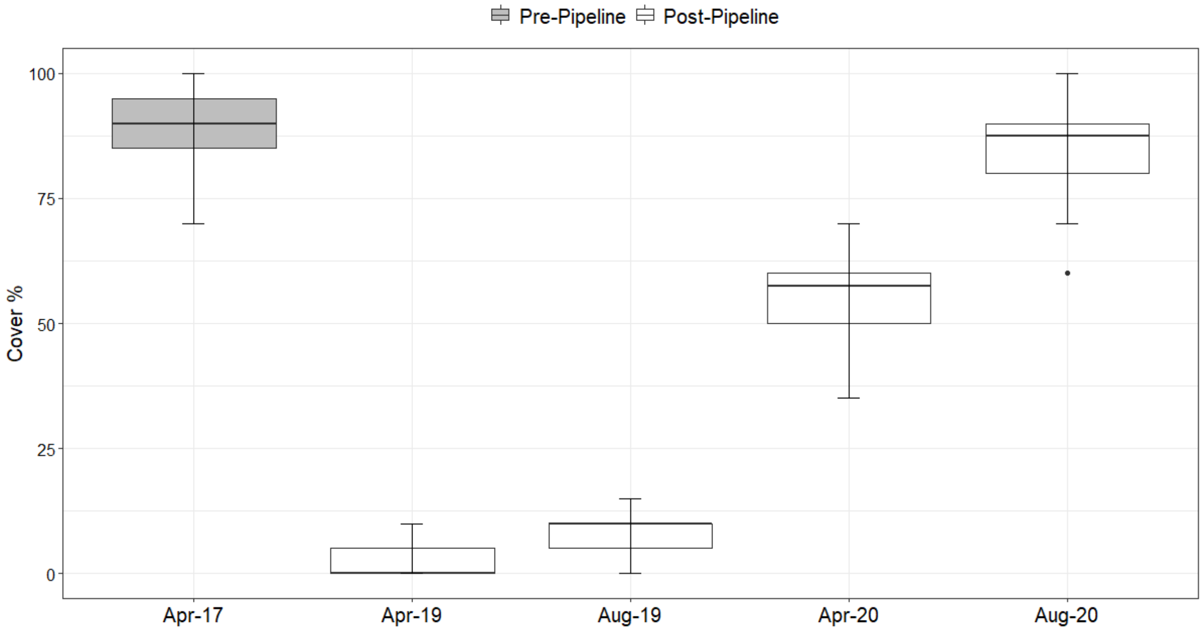

Figure S2. Differences in cover percentage between the five sampling events (pre-pipeline construction in grey and post-pipeline construction in white). Letters above the boxes indicate significant differences based on Tukey's HSD at the $p < 0.05$ threshold.

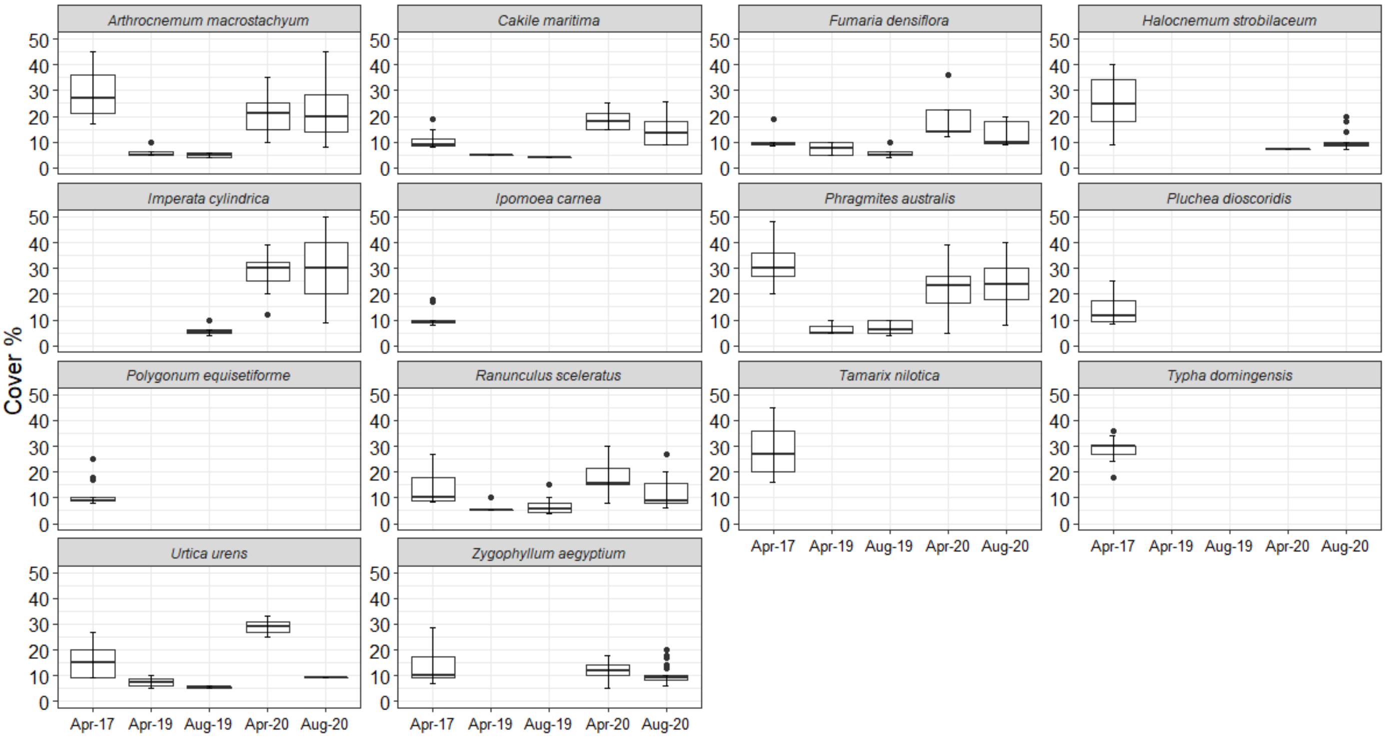

Figure S3. Differences in total species cover percentages per plot for the five sampling events (before and after the megaproject).

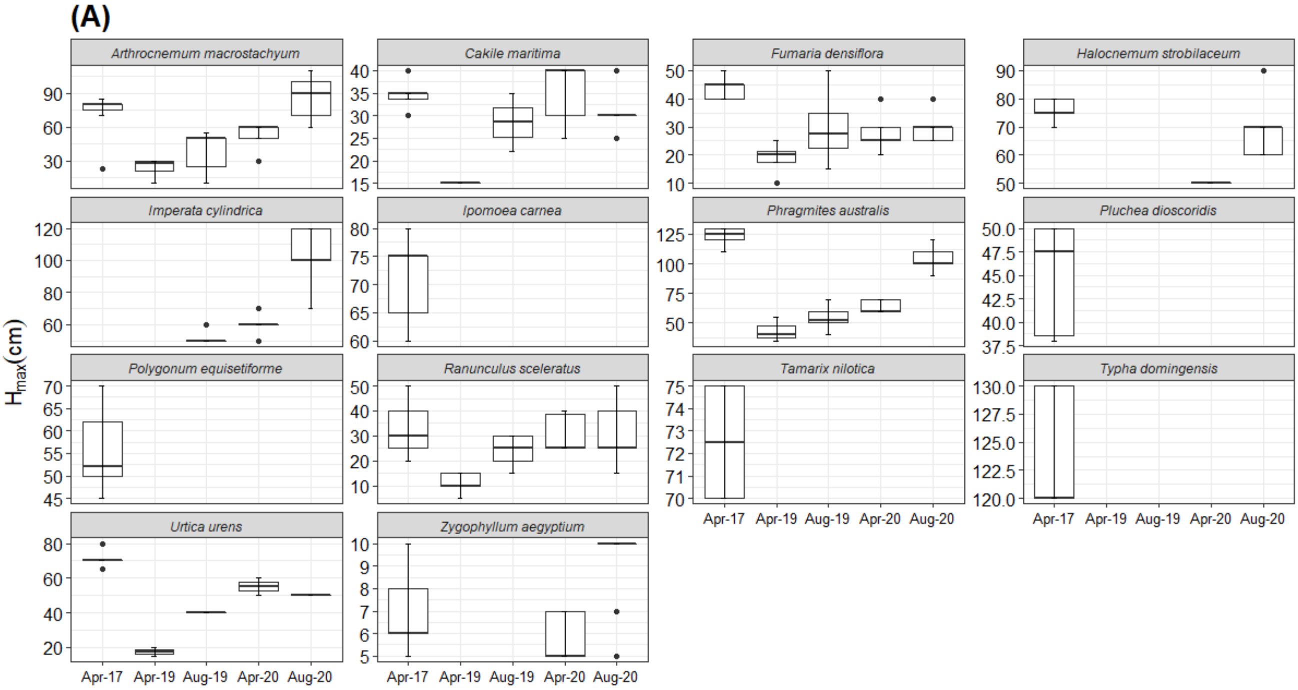

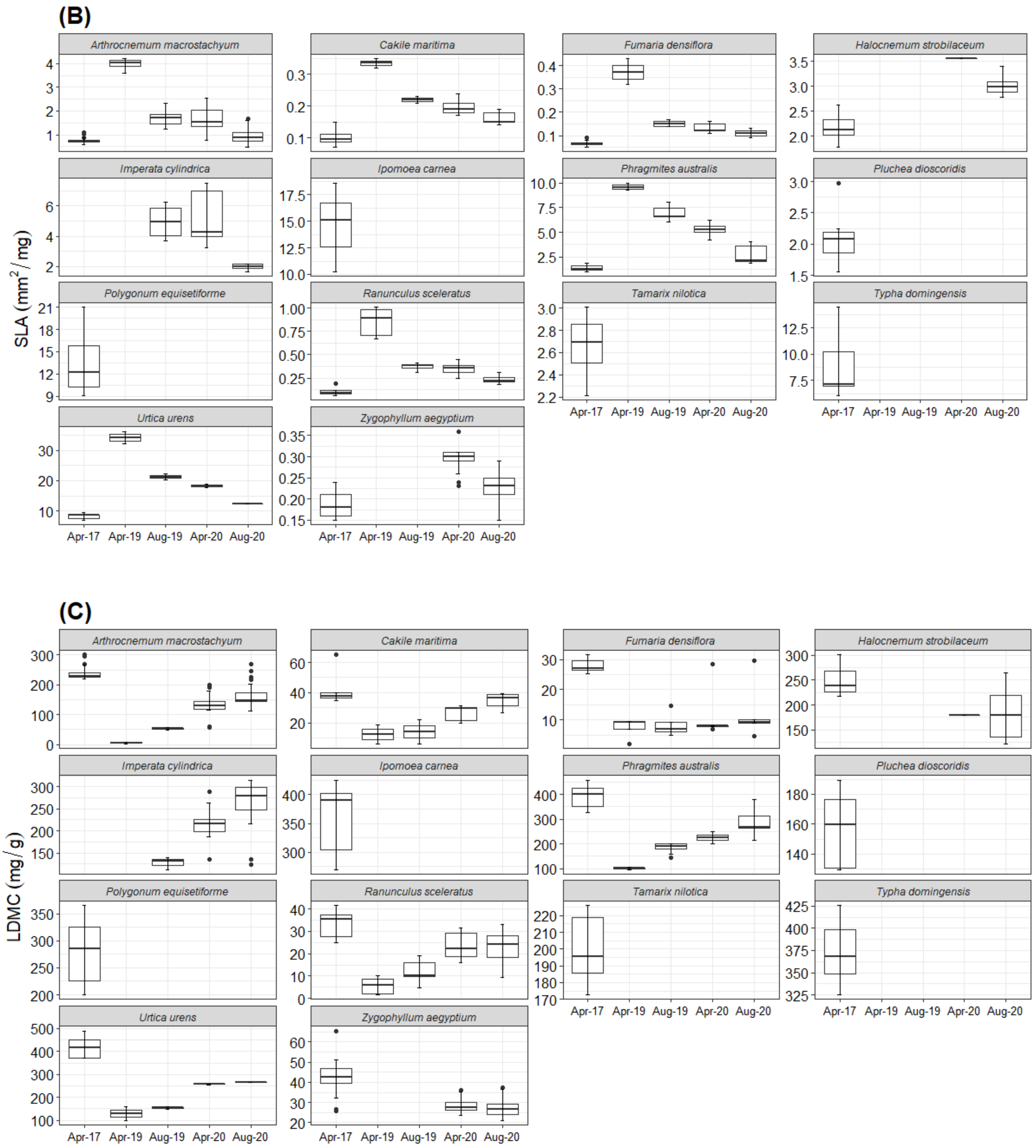

Figure S4. Differences of plant functional traits between species for the five sampling events (before and after the project) in (**A**) maximum plant height (H_{max}), (**B**) specific leaf area (SLA), and (**C**) leaf dry matter content (LDMC).

Table S1. ANOVA output for differences in maximum plant height (H_{max}), specific leaf area (SLA), and leaf dry matter content (LDMC) between the 14 recorded species for the five sampling events.

Species	H_{max}			SLA			LDMC		
	F	df	*P*	*F*	df	*P*	*F*	df	*P*
Arthrocnemum macrostachyum (Moric.) C, Koch	40.5	118	<0.001	121.3	118	<0.001	119.3	118	<0.001
Cakile maritima Scop.	6.165	17	<0.01	42.71	17	<0.001	7.97	17	<0.001
Fumaria densiflora DC.	9.74	23	<0.001	133.9	23	<0.001	13.73	23	<0.001
Halocnemum strobilaceum (Pall.) Bieb.	17.56	35	<0.001	86.91	35	<0.001	15.4	35	<0.001
Imperata cylindrica (L.) P. Beauv.	203	81	<0.001	114.5	81	<0.001	57.25	81	<0.001
Ipomoea carnea Jacq.	-	-	-	-	-	-	-	-	-
Phragmites australis (Cav.) Trin. ex Steud.	335.1	80	<0.001	282.9	80	<0.001	111	80	<0.001
Pluchea dioscoridis (L.) DC.	-	-	-	-	-	-	-	-	-
Polygonum equisetiforme Sibth. & Sm.	-	-	-	-	-	-	-	-	-
Ranunculus sceleratus L.	15.2	91	<0.001	310.6	91	<0.001	51.43	91	<0.001
Tamarix nilotica (Ehrenb.) Bunge	-	-	-	-	-	-	-	-	-
Typha domingensis Pers.	-	-	-	-	-	-	-	-	-
Urtica urens L.	66.02	12	<0.001	186.1	12	<0.001	31.96	12	<0.001
Zygophyllum aegyptium A. Hosny	67.33	97	<0.001	81.83	97	<0.001	93.64	97	<0.001

Table S2. The coefficient of variation (cv) of the maximum plant height (H_{max}), specific leaf area (SLA), and leaf dry matter content (LDMC) for each species at each of the five sampling events (before and after the project).

Species	Date	The Coefficient of Variation (cv)		
		H_{max}	SLA	LDMC
Arthrocnemum macrostachyum	April 2017	24.86	16.25	8.94
Arthrocnemum macrostachyum	April 2019	39.85	6.81	51.06
Arthrocnemum macrostachyum	August 2019	41.99	19.45	5.31
Arthrocnemum macrostachyum	April 2020	12.10	27.65	28.10
Arthrocnemum macrostachyum	August 2020	21.18	27.99	22.42
Cakile maritima	April 2017	9.32	25.01	24.66
Cakile maritima	April 2019	0.00	6.33	74.13
Cakile maritima	August 2019	32.25	6.43	81.50
Cakile maritima	April 2020	20.20	14.01	20.60
Cakile maritima	August 2020	17.67	13.38	15.79
Fumaria densiflora	April 2017	7.76	19.16	7.73
Fumaria densiflora	April 2019	33.55	12.85	49.05
Fumaria densiflora	August 2019	49.07	9.83	50.85
Fumaria densiflora	April 2020	27.08	16.42	78.91
Fumaria densiflora	August 2020	20.41	14.37	78.56
Halocnemum strobilaceum	April 2017	4.92	10.53	11.14
Halocnemum strobilaceum	April 2019	-	-	-
Halocnemum strobilaceum	August 2019	-	-	-
Halocnemum strobilaceum	April 2020	-	-	-
Halocnemum strobilaceum	August 2020	10.89	5.85	26.10
Imperata cylindrica	April 2017	-	-	-
Imperata cylindrica	April 2019	-	-	-
Imperata cylindrica	August 2019	8.11	19.99	7.12
Imperata cylindrica	April 2020	7.94	30.21	15.60
Imperata cylindrica	August 2020	12.43	8.05	16.51
Ipomoea carnea	April 2017	9.48	17.07	13.97
Ipomoea carnea	April 2019	-	-	-
Ipomoea carnea	August 2019	-	-	-
Ipomoea carnea	April 2020	-	-	-

Ipomoea carnea	August 2020	-	-	-
Phragmites australis	April 2017	6.32	19.79	11.14
Phragmites australis	April 2019	24.02	4.03	4.45
Phragmites australis	August 2019	14.11	9.55	9.34
Phragmites australis	April 2020	7.55	9.13	5.95
Phragmites australis	August 2020	6.56	29.40	14.94
Pluchea dioscoridis	April 2017	12.79	17.98	15.24
Pluchea dioscoridis	April 2019	-	-	-
Pluchea dioscoridis	August 2019	-	-	-
Pluchea dioscoridis	April 2020	-	-	-
Pluchea dioscoridis	August 2020	-	-	-
Polygonum equisetiforme	April 2017	12.95	23.96	18.88
Polygonum equisetiforme	April 2019	-	-	-
Polygonum equisetiforme	August 2019	-	-	-
Polygonum equisetiforme	April 2020	-	-	-
Polygonum equisetiforme	August 2020	-	-	-
Ranunculus sceleratus	April 2017	25.76	37.64	16.14
Ranunculus sceleratus	April 2019	30.00	15.59	59.39
Ranunculus sceleratus	August 2019	22.46	8.85	40.19
Ranunculus sceleratus	April 2020	22.42	15.07	24.15
Ranunculus sceleratus	August 2020	31.86	16.52	28.55
Tamarix nilotica	April 2017	3.69	11.31	10.61
Tamarix nilotica	April 2019	-	-	-
Tamarix nilotica	August 2019	-	-	-
Tamarix nilotica	April 2020	-	-	-
Tamarix nilotica	August 2020	-	-	-
Typha domingensis	April 2017	3.70	27.66	8.72
Typha domingensis	April 2019	-	-	-
Typha domingensis	August 2019	-	-	-
Typha domingensis	April 2020	-	-	-
Typha domingensis	August 2020	-	-	-
Urtica urens	April 2017	6.63	11.98	11.31
Urtica urens	April 2019	20.20	8.58	32.56
Urtica urens	August 2019	0.00	7.16	4.44
Urtica urens	April 2020	12.86	4.83	1.32
Urtica urens	August 2020	-	-	-
Zygophyllum aegyptium	April 2017	25.69	15.43	17.25
Zygophyllum aegyptium	April 2019	-	-	-
Zygophyllum aegyptium	August 2019	-	-	-
Zygophyllum aegyptium	April 2020	17.20	13.84	11.08
Zygophyllum aegyptium	August 2020	9.42	13.47	14.57
